# Acceleration of the sliding movement of actin filaments with the use of a non-motile mutant myosin in *in vitro* motility assays driven by skeletal muscle heavy meromyosin

**DOI:** 10.1371/journal.pone.0181171

**Published:** 2017-07-24

**Authors:** Kohei Iwase, Masateru Tanaka, Keiko Hirose, Taro Q. P. Uyeda, Hajime Honda

**Affiliations:** 1 Department of Bioengineering, Nagaoka University of Technology, Nagaoka, Japan; 2 Biomedical Research Institute, National Institute of Advanced Industrial Science and Technology, Tsukuba, Ibaraki, Japan; Semmelweis Egyetem, HUNGARY

## Abstract

We examined the movement of an actin filament sliding on a mixture of normal and genetically modified myosin molecules that were attached to a glass surface. For this purpose, we used a *Dictyostelium* G680V mutant myosin II whose release rates of Pi and ADP were highly suppressed relative to normal myosin, leading to a significantly extended life-time of the strongly bound state with actin and virtually no motility. When the mixing ratio of G680V mutant myosin II to skeletal muscle HMM (heavy myosin) was 0.01%, the actin filaments moved intermittently. When they moved, their sliding velocities were about two-fold faster than the velocity of skeletal HMM alone. Furthermore, sliding movements were also faster when the actin filaments were allowed to slide on skeletal muscle HMM-coated glass surfaces in the motility buffer solution containing G680V HMM. In this case no intermittent movement was observed. When the actin filaments used were copolymerized with a fusion protein consisting of *Dictyostelium* actin and *Dictyostelium* G680V myosin II motor domain, similar faster sliding movements were observed on skeletal muscle HMM-coated surfaces. The filament sliding velocities were about two-fold greater than the velocities of normal actin filaments. We found that the velocity of actin filaments sliding on skeletal muscle myosin molecules increased in the presence of a non-motile G680V mutant myosin motor.

## Introduction

Actin filaments are one of the major and ubiquitous cytoskeletal filaments that play multiple important functions in various types of cells, including the contraction of muscles driven by the sliding movement of actin filaments along myosin molecules within a series of sarcomeres. This type of motion can be reconstituted *in vitro* by allowing actin filaments to move over surfaces coated with myosin molecules, then visualized and quantified under a fluorescent microscope [1, 2]. The movements are unidirectional and the speeds remain largely invariable under constant conditions. Moreover, the same skeletal muscle actin filaments move at different velocities on surfaces coated with different types of myosin, leading to the notion that the velocity of an actin filament is primarily determined by the property of driving myosin molecules [3]. Indeed, in the prevailing swinging lever arm theory, the sliding velocity is primarily determined by the step size divided by the duration of the strongly bound state [4].

However, some cases strongly suggest the idea that not only the properties of myosin, but also the dynamic properties of actin filaments, may play significant roles in determining sliding velocities. For example, actin filaments treated with glutaraldehyde [5] and those cross-linked between neighboring protomers along a long pitch helix [6] are unable to slide on muscle HMM-coated surfaces. Since the two types of modified actin filaments are able to bind strongly to myosin motors and stimulate their ATPase activity, their effects are difficult to reconcile in the light of the simple lever arm hypothesis. In the lever arm hypothesis, there are only two roles of actin filaments: one is to stimulate phosphate release from myosin motor carrying ADP and phosphate, and the other is to provide a foothold for the myosin motor to maintain tension [7, 8]. Rather, the above mentioned results strongly support the idea that myosin-induced conformational changes of actin filaments, which have been demonstrated in various biophysical measurements [9–12], are critically important for proper actomyosin motility [13]. This perspective gains its support from detailed observations of an *in vitro* actomyosin motility assay involving normal actin filaments. For example, at a low ATP concentration, mechanical distortions were found to have major roles in the smooth sliding motion of filaments [14]. Furthermore, a cross-correlation study revealed that slowly moving filaments are apparently more rigid than rapidly moving filaments [15]. These and other reports [16] strongly suggest that the sliding movement involves not only myosin motor activity but also the dynamic behavior of actin filaments.

Intriguingly, the inclusion of non-native myosin substrates, such as Mg-GTP [17] or AMPPNP [18], in the motility assay buffer enhances the sliding velocity of actin filaments *in vitro*. However, few studies have reported acceleration of the sliding movement of an actin filament by modulating the dynamic properties of the system. We speculated that myosin-induced conformational changes in actin filaments, if any, would likely accelerate the movement of actin filaments by somehow augmenting actin-myosin interactions. From such a viewpoint, we turned our attention to a G680V mutant myosin II from *Dictyostelium* [19]. G680V mutant myosin was reported to possess strong affinity for either ADP and phosphate [20] or ADP [21]. Even though the chemical or structural state of G680V mutant myosin bound with actin filaments is controversial, we can say for certain that G680V heads remain bound to actin filaments for a long period of time, even in the presence of normal concentrations of ATP, in a conformation that is distinct from the rigor of crossbridges. Consequently, G680V mutant myosin is hardly able to drive the movement of actin filaments on its own *in vitro* [19]. In this report, we investigated the behavior of filaments when multiple types of HMM act on one filament. We observed, in three *in vitro* motility assays, that the interaction of actin filaments with a small number of G680V myosin motors significantly enhanced the velocity of actin filaments sliding on a skeletal muscle HMM surface, which is consistent with the ideas elaborated above.

## Materials and methods

### Proteins and reagents

Sodium ATP was purchased from Sigma Co. Ltd. Other reagents were of analytical grade from Wako Pure Chemicals. Proteins from rabbit fast skeletal muscle were prepared as described in a previous report [22]. G680V full length *Dictyostelium* myosin II was purified as described previously [19]. The G680V mutation was introduced into the coding sequence of *Dictyostelium* HMM [22] and into the *Dictyostelium* acto-S1dC fusion protein [23], and the recombinant protein of each was purified as explained in each study. All proteins were rapidly frozen and stored in liquid nitrogen, and thawed before use. Three different preparations of G680V full length myosin were examined in preliminary experiments and the presented data was obtained using the fourth, and last, preparation. In all cases, G680V showed essentially the same results, i.e., the addition of a small amount of the mutant myosin increased the sliding velocity. Single batches of G680V HMM and acto-G680V S1dC fusion protein were prepared and used in this study. *N*-ethylmaleimide (NEM) treatment of skeletal muscle S1 was performed according to the method of Sekine [24] and used within a week after its preparation. This study was reviewed and approved by the Nagaoka University of Technology Research Ethics Committee.

Fast skeletal muscle actin or a mixture of the muscle actin and G680V acto-S1dC fusion protein was allowed to polymerize at 1.56 μM for 60 min by adding tetramethylrhodamine-phalloidin at a molar ratio of 1:1 under the following conditions: 25 mM imidazole-HCl (pH = 7.4), 25 mM KCl, 4.0 mM MgCl_2_, 2.0 mM adenosine triphosphate (ATP), and 50 mM dithiothreitol (DTT).

### Microscopic observations

The motility of actin filaments in this study was assayed as described previously [18], except when mentioned otherwise. Glass slides (Matsunami 24×50 mm, 0.12 mm thickness) were dipped into piranha solution, 1:2 mixture of sulfuric acid and hydrogen peroxide, for 20 s followed by four immediate washes with 500 mL of double distilled water (DDW). Slides were air dried for 60 min and subjected to hydrophobic treatment by dipping into 1.0% of nitrocellulose dissolved in isoamyl acetate for 10 s, followed by a brief rinse with the same solvent, and dried in air. The slides were stored in a clean chamber and used within one week. A flow chamber with 0.1 mm thickness was prepared as follows: polypropylene double-sticky tape (NITOMZ No.539R 50 mm×20 m J0850) of 0.1 mm thickness was cut into 2.0×20 mm size pieces. Two pieces of double-sided sticky tape were attached to both edges of an untreated cover slip (Matasunami 18×18 mm, 0.12 mm thickness) and the cover slip was placed on the center of the nitrocellulose-treated glass slide so that the edge of the tape was parallel to the glass slide. Reaction solutions were sequentially added drop-wise every 30 s at one end of the chamber and suctioned out by filter paper at the opposite end. Four solutions were added in the following order: solution 1 (25 mM imidazole-HCl (pH = 7.4), 25 mM KCl, 4.0 mM MgCl_2_, 50 mM DTT), solution 2 (0.25 μM HMM or a mixture of HMM and G680V myosin in solution 1), solution 3 (30 mg/mL BSA in solution 1), solution 4 (0.5 μg/mL labeled actin filament in solution 1). The sliding reactions were initiated by adding solution 5 (2.0 mM Mg-ATP in solution 1) to the flow chamber on the microscopic stage. The experiments were performed at 26^°^C with less than 30% relative humidity.

When we immobilized skeletal muscle HMM and G680V full length myosin on the surface, the two types of myosins were premixed in solution 1 at a given ratio of concentrations while maintaining the total concentration at 0.25 μM before introducing them into the flow chamber. Therefore, the ratio of the two types of myosin molecules on the surface was not specified. However, in order to induce continuous motility of actin filaments on surfaces coated with full length *Dictyostelium* myosin, 0.5~1.0 μM was required [2], whereas 0.25 μM of skeletal muscle HMM was sufficient. We thus speculate that the affinity of full length *Dictyostelium* myosin for nitrocellulose surfaces is similar to or less than that of skeletal muscle HMM, and that the ratio of surface density of full length *Dictyostelium* G680V myosin and skeletal muscle HMM is similar to or less than that in the solution.

Sliding movements were observed under a fluorescence microscope (NIKON ECLIPSE TE200-U) and video images were recorded by a CCD camera (Hitachi Kokusai Electric KP-E500) and an image-capture board (DFG/SV1) and stored on a personal computer. The resolution of images was 640×480 pixels with 30 frames/s. The accurate position of either end of a filament was difficult to determine due to fluctuation caused by a thermal bending motion. Thus, the center of a moving filament was determined manually every 0.1 s for 10 s [14] by using Image-J software. The resulting position data were subject to velocity calculations. Displacements less than 0.2 μm within 10 s were eliminated from further data processing to reduce experimental errors. Each data point was calculated from velocities of 100 filaments (20 filaments each from 5 independent experiments). Filaments longer than 4 μm were excluded from the analysis because they tended to follow curved paths causing experimental errors in determining the center of the filament. The distribution of filament lengths, which fitted well with a Gaussian distribution, had an average length of 1.72 μm, S.D. = 0.43.

Samples for electron microscopic observations were stained negatively in a conventional manner and observed by an FEI Tecnai™ transmission electron microscope (TECNAI F20) with a scanning system (Carl Zeiss, PHODIS SC).

## Results

### G680V myosin fixed on the glass surface

**Fig 1:**
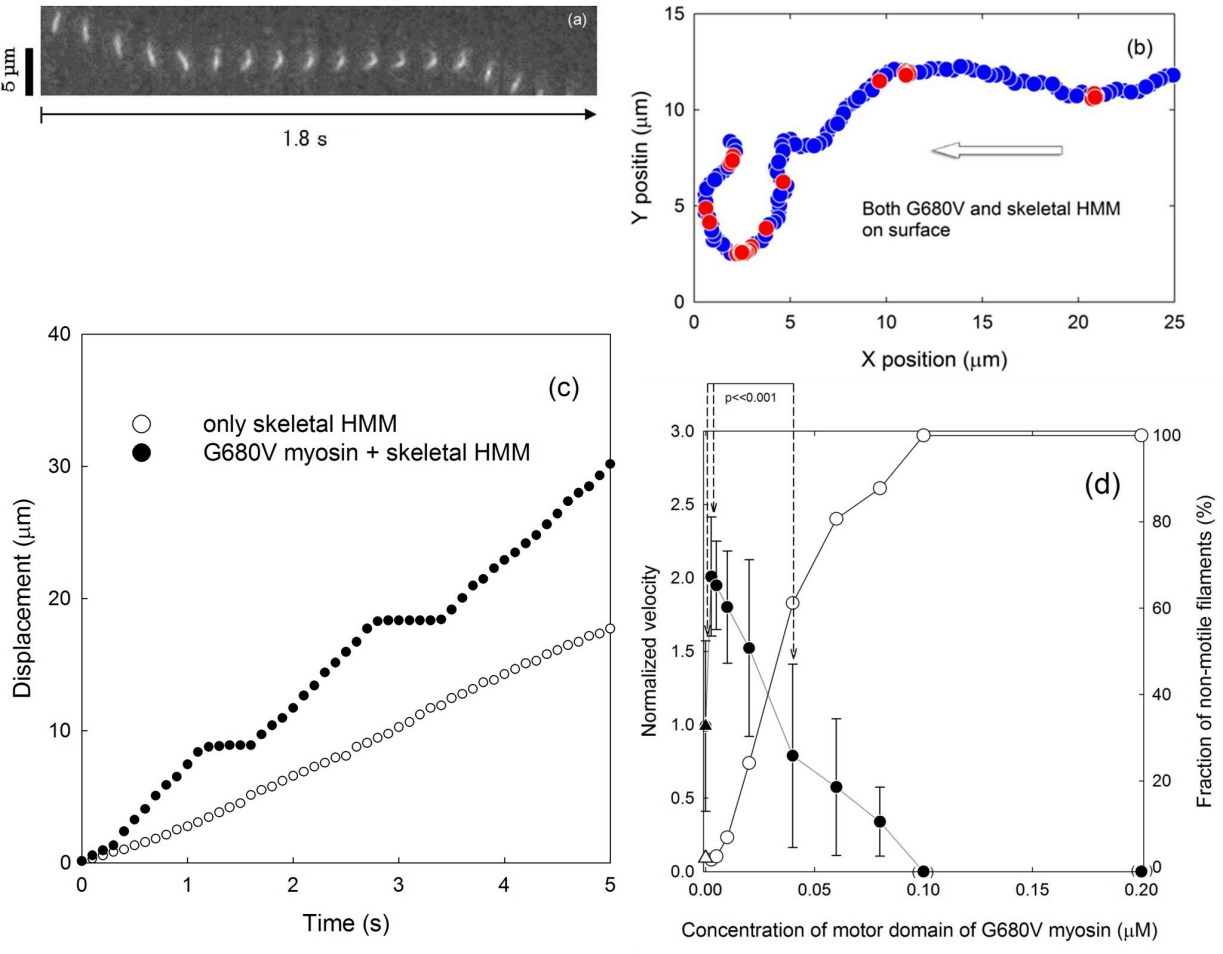
Motion of filaments driven by both G680V myosin and skeletal HMM on glass surfaces. A set of sequential photographs of a representative moving filament driven by a mixture of skeletal HMM and G680V myosin immobilized on a glass surface (a). The concentration of skeletal HMM+G680V myosin in the perfusion solution was 0.25 μM with 0.25% (w/w) G680V myosin. Each photograph was taken at 0.1 s intervals. A trajectory of this moving filament is shown in panel (b), in which red and blue symbols represent instantaneous velocities below and over 5.0 μm/s, respectively. Horizontal white arrow indicates the direction of the overall motion. In panel (c), the integrated displacements of this filament are shown with open symbols while the closed symbols show those in the absence of G680V myosin. The effects of G680V myosin concentration on the velocity (closed symbols) and on the ratio of moving filaments (open symbols) are shown in panel (d). The average velocities of filaments (n = 100, i.e. 20 filaments from 5 independent experiments) are shown. Error bars represent standard deviations (n = 100). When the identified filaments were not moving, the corresponding data for the normalized velocity were marked as zeros within brackets. The triangle symbols near the left vertical axis are for the data in the absence of G680V myosin. The difference between the sliding velocities in the presence of 0.01 μM G680V motor domain and those in the presence of 0.04 μM or in the absence of G680V motor domain were statistically significant by a student’s *t*-test (p<<0.001).

In the first experiment, G680V full-length myosin was mixed with skeletal muscle HMM at controlled ratios while maintaining total myosin concentration constant at 0.25 μM in assay solution 1. Full length G680V myosin, rather than G680V HMM, was used because *Dictyostelium* HMM is hardly immobilized on nitrocellulose surfaces in contrast to myosin II [2]. The flow chamber was washed with solution 1 twice to remove free myosin and HMM in solution. Fig 1(A) shows a sequence of photographs of a moving filament with a long pause in the middle of the time interval. The trajectory of this filament with 0.1 s intervals (b) and the time-dependent accumulation of the displacements (c) are also shown in Fig 1. As shown in Fig 1(C), typical trajectories consisted of two easily distinguishable phases, pauses and a moving phase with a relatively constant speed.

Differences in the sliding velocities normalized against those in the absence of G680V myosin, and the fraction of non-motile filaments were plotted against the concentrations of G680V myosin in the initial mixture (Fig 1(D)). The velocity of each filament was calculated by retrieving the average distances moved in 0.1 s during the moving phases, while excluding non-motile filaments and motile filaments during pauses in movement. Notably, the normalized velocity when the concentration of G680V myosin in the initial myosin mixture was 0.01 μM was two-fold faster than that in the absence of G680V myosin (p<0.001 using a student’s *t*-test). No sliding movement took place when the concentration of G680V myosin in the initial mixture was higher than 50%, i.e. 0.125 μM, which was consistent with previous observations [19].

### Myosin motor molecules added to the motility assay solution

#### Addition of G680V HMM to the motility assay solution

**Fig 2:**
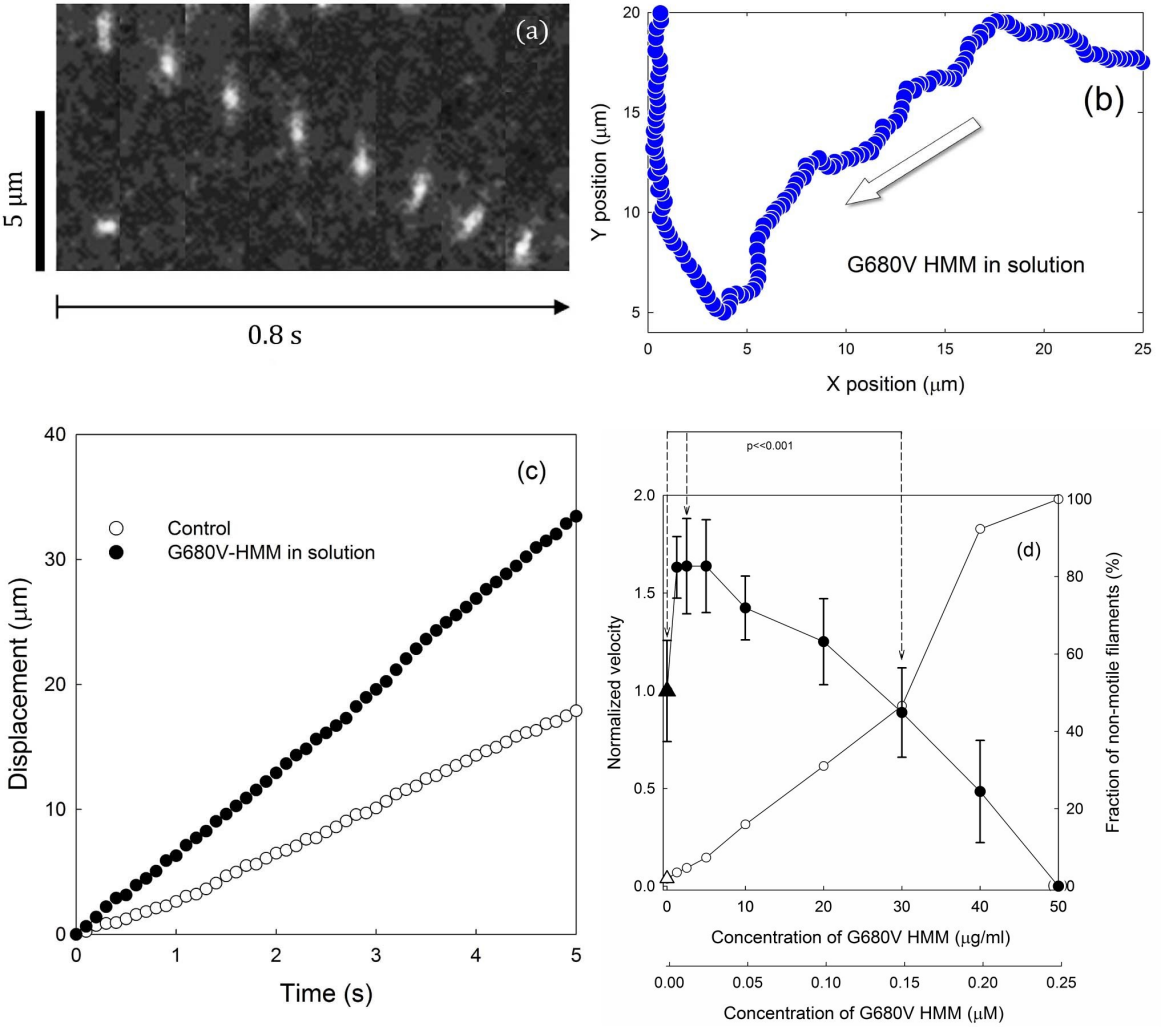
Motion of actin filaments driven by skeletal HMM on glass surfaces in the presence of G680V HMM in the assay solution. Sequential micrographs of a moving filament are shown in panel (a). A short filament was observed to move downward with a nearly constant velocity. The trajectory of this moving filament is shown in panel (b) with an arrow representing the overall direction of the movement. The integrated displacements of filament motion in the presence of 0.001 μM of G680V HMM in the assay solution 5 are shown (filled symbols) in panel (c), in which open symbols indicate the control in the absence of G680V HMM. Panel (d) displays the effects of G680V concentration on the velocity (filled symbols) and the fraction of non-motile filaments (open symbols). The average velocities of filaments (n = 100, i.e. 20 filaments from 5 independent experiments) are shown. Bars indicate standard deviations. The sliding velocities in the presence of 0.02 μM G680V motor domain were compared to those in the absence and presence of 0.15 μM G680V motor domain. The differences were statistically significant by a student’s *t*-test (p<<0.001).

After conventional preparation of the flow chamber with skeletal muscle HMM and actin filaments, solution 5 containing ATP and G680V HMM at various concentrations was perfused, to start movement. Photographs of sequential segments of this movement (a) and the trajectory of a filament position (b) and the total displacements (c) in the presence of 0.005 μM G680V HMM are shown in Fig 2. The velocity under this condition, which was read from the slope of the filled symbols in (c), was about two-fold faster than in the absence of G680V HMM, and was similar to that obtained from the moving phases in Fig 1(C).

Sliding velocities (a) and the fraction of moving filaments (b) plotted against the concentrations of G680V HMM in the assay solution are shown in Fig 2(D). Velocities were normalized against velocities in the absence of G680V HMM in the motility assay solution. The velocities of filaments in the presence of less than 0.025 μM G680V HMM increased by about 1.7 fold. In the presence of higher concentrations of G680V HMM, actin filaments either stopped moving or dissociated from the surface.

#### Addition of skeletal muscle S1 or NEM-S1 to the motility assay solution

**Fig 3:**
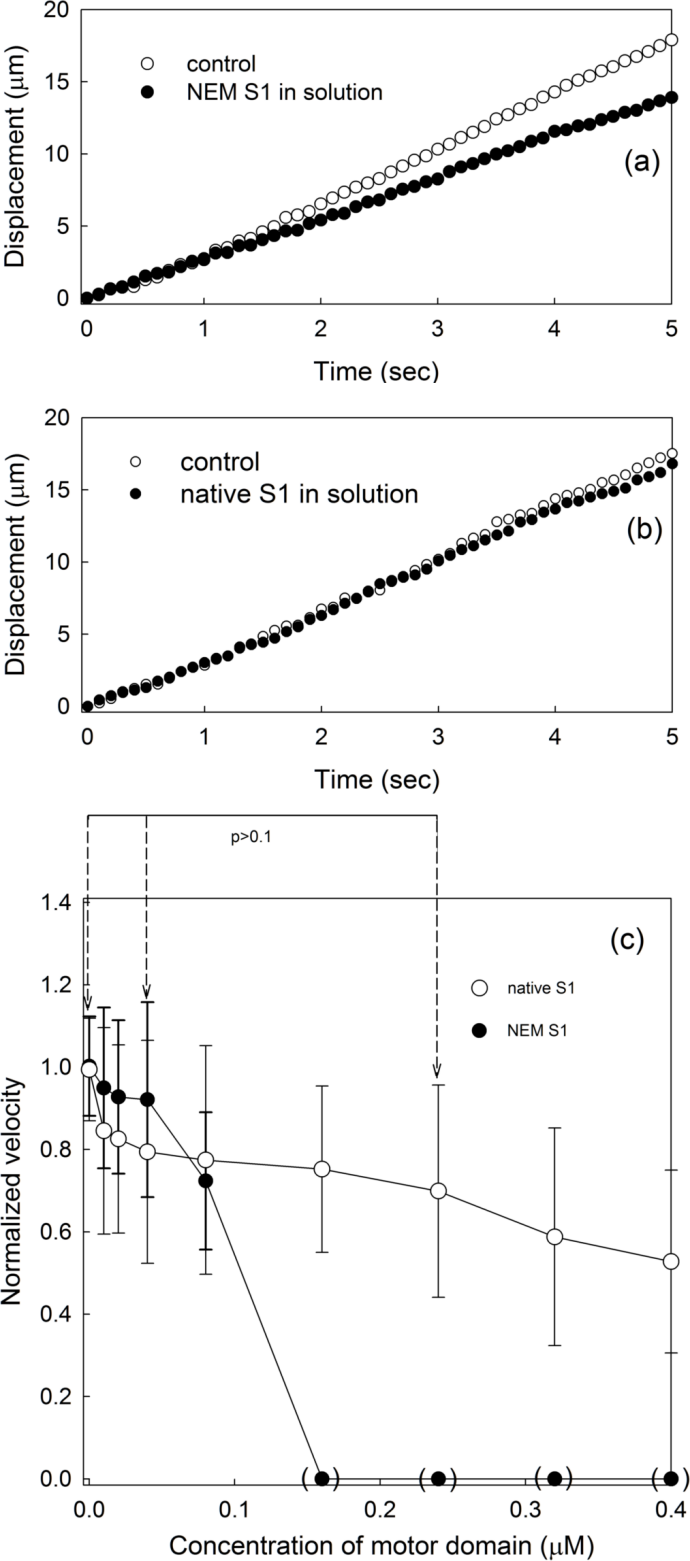
Motion of actin filaments in the presence of skeletal NEM-S1 in the motility assay solution. The integrated displacements of filaments in the absence (open symbols), and presence of NEM-S1 (filled symbols) in the motility assay solution are shown in panel (a). No apparent differences were detected. The integrated displacements of filaments in the absence (open symbols) and presence of native skeletal muscle S1 (filled symbols) in solution are shown in panel (b). The concentration of native or NEM S1 was 0.0125 μM. In panel (c), the average velocities of the filaments in the presence of either NEM-S1 (filled symbols) or native S1 (open symbols) have been normalized. Bars indicate standard deviations for 100 filaments (n = 100, i.e. 20 filaments from 5 independent experiments). If moving filaments were not detected, the corresponding data for the normalized velocities are denoted as zeros within brackets. Observations were completed within 5 min after the addition of the assay buffer containing the respective S1. The sliding velocities in the presence of 0.04 μM motor domain were compared to those in the absence and presence of 0.24 μM motor domain for both cases in the presence of native S1 or NEM-S1. However, there were no statistically significant differences by a student’s *t*-test (p>0.1).

NEM treatment of S1 is known to increase the binding affinity of S1 to actin filaments, even in the presence of ATP [25]. If the acceleration by G680V HMM is simply caused by binding of myosin motors with a high affinity, then NEM-HMM in the motility assay solution is also expected to increase the velocity of filaments. To test this possibility, moving velocities were measured when NEM-treated S1 (NEM-S1) was included in the motility assay buffer. As a control experiment, native skeletal muscle S1 was included in the motility assay buffer. Fig 3 shows the increase in displacements in the presence of NEM-S1 (a) and native S1 (b) indicated by filled symbols. In both panels, displacements in the absence of S1 in the assay solution are shown in open symbols, as the control. No significant differences were detected in either experiment. The normalized velocities in both cases are shown in panel (c). NEM-S1 did not significantly accelerate or slow down filament movement but prevented motion when added at concentrations higher than 0.1 μM, as indicated by filled symbols in brackets in panel (c).

### Incorporation of Acto-G680V S1dC chimera molecules into actin filaments

#### Motility of the copolymers

**Fig 4:**
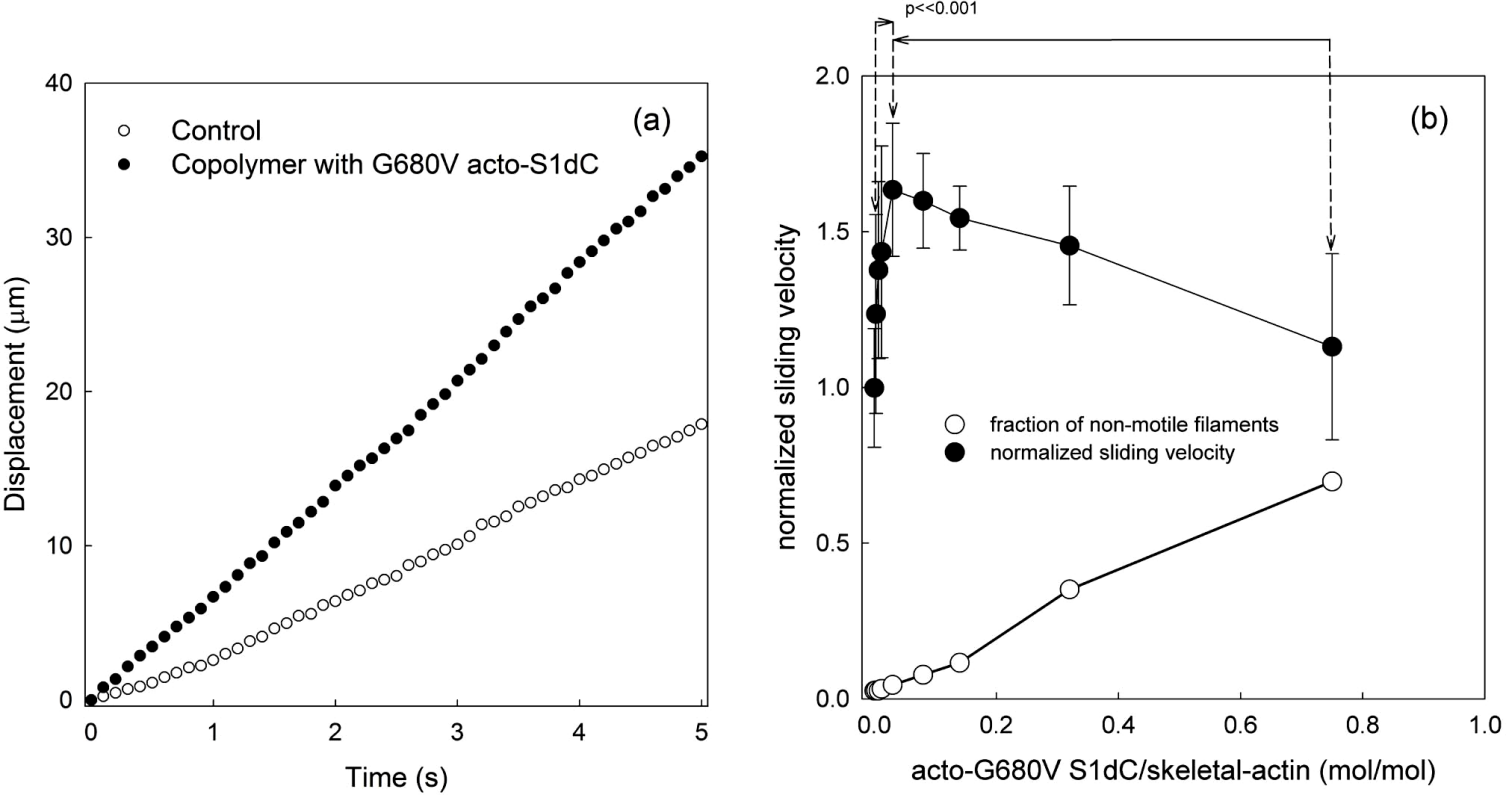
Motion of copolymer of skeletal muscle actin and acto-G680V S1dC. Panel (a) displays integrated displacements of a control actin filament (open symbols) and a copolymer of skeletal muscle actin and acto-G680V S1dC (1/30 mol/mol) (filled symbols). In panel (b), the normalized sliding velocities (filled symbols) and the fraction of non-motile filaments (open symbols, n = 100, i.e. 20 filaments from 5 independent experiments) are shown as a function of the mixing ratio of acto-G680V S1dC and skeletal muscle actin in the polymerization solution. The sliding velocities of the filaments with a mixing ratio of 0.05 (mol/mol) of acto-G680V S1dC were compared to those in the absence of and presence of a 0.8 (mol/mol) mixing ratio. The differences were statistically significant based on a student’s *t*-test (p<<0.001).

Acto-G680V S1dC was copolymerized with skeletal muscle G-actin at a molar ratio from 0.0 to 0.8. The resulting copolymer filaments were subjected to conventional *in vitro* motility assays. As demonstrated in Fig 4, copolymerization of skeletal muscle actin with 1/30 acto-G680V S1dC significantly accelerated movement on surfaces coated with skeletal muscle HMM. The accelerated velocity was similar to that observed in the two earlier experiments (Figs 1 and 2).

#### TEM observations

**Fig 5:**
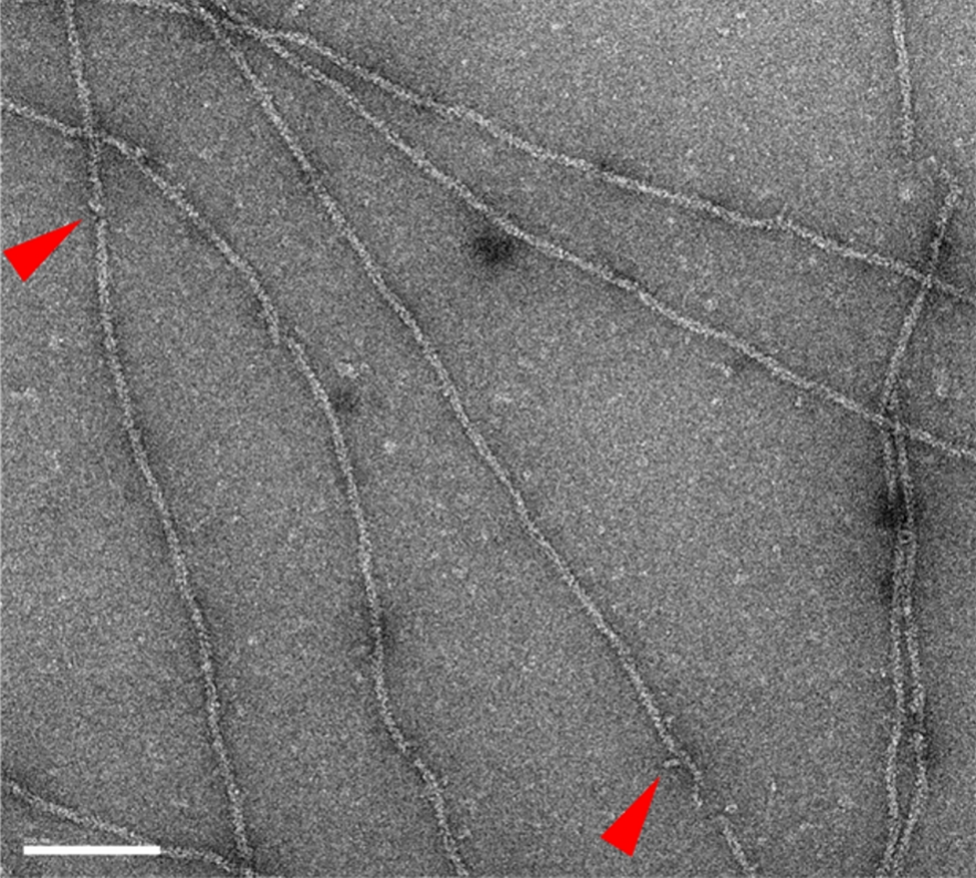
An electron micrograph of copolymers of skeletal actin and acto-G680V S1dC (30:1). Copolymers containing 1/30 (mol/mol) of acto-G680V S1dC were identified. Red arrows indicate protrusions which presumably represent the S1dC moiety of acto-G680V S1dC. Bar indicates 100 nm.

Copolymers containing 1/30 of acto-G680V S1dC that were negatively stained with uranyl acetate were identified by transmission electron microscopy (Fig 5). Arrows indicate the protrusions supposed to be the S1dC moiety of acto-G680V S1dC molecules.

## Discussion

### Effect of G680V myosin coexisting with skeletal HMM on a glass surface

We found that the presence of trace amounts of non-motile G680V mutant myosin on skeletal HMM-coated surfaces significantly accelerated the movement of actin filaments *in vitro*. There may be three possible explanations for the faster movement of actin filaments. First, the most trivial explanation, is that the small number of G680V full length myosin on the surface inhibited the movement of the filament away from the surface, and promoted the sliding movement much the same way as inclusion of methylcellulose helps sliding movement when the interaction between the actin filaments and the surface is too weak to maintain the continuous sliding movement. However, this possibility is unlikely because inclusion of methylcellulose does not accelerate the movement above the speed attained under appropriate conditions without methylcellulose [4]. Second, energy for rapid movement might have accumulated during pauses when part of the filament was attached to a G680V molecule and motion was suppressed. The energy stored as mechanical strain causing either compressive, elongating or torsional deformations within the filament [14], may be released during the following moving phase to accelerate the movement. When over 0.25 nM G680V full length myosin was added to the myosin solution to coat the surface of the flow chamber, strong binding between G680V-domain and actin filament overcame the sliding force generated by intact HMM molecules, which was consistent with previous observations [19]. In this scenario, a part or parts of the actin filament must be anchored to the surface via G680V myosin motors. A third possibility is that rapid movement was caused by changes in the state or conformation of the filament induced by the continuous association and dissociation of G680V myosin motors. The following two experiments were performed to determine which of these three possibilities is more likely, by testing whether the faster movement of actin filaments depended on the anchoring of a part of the filament to the substrate via a G680V myosin motor(s).

### Effect of myosin fragments in solution

First, in order to test the requirement of anchoring filaments, we added G680V HMM to the motility assay solution that contained ATP, so that the G680V myosin motors were not anchored to the glass surfaces. The photographs and trajectory shown in Fig 3 indicate that the sliding motions were uniform and about 1.7-fold faster than those driven by skeletal-HMM alone. Since the G680V HMM molecules were free in solution, they could not produce contractile or protractile distortions to the moving actin filaments. The observed acceleration is due to the actin filaments interacting with G680V HMM molecules, but anchoring filaments to the surface via G680V myosin motors is not only unnecessary, it may have inhibited faster movement since there were pauses that interrupted faster movements when G680V myosin was immobilized to the surface. This result ruled out the first two possibilities mentioned above, and led us to conclude that G680V myosin motors accelerate the actin movements propelled by skeletal HMM by changing the state or conformation of the actin filaments.

When NEM-S1, which is known to possess a strong affinity for actin filaments in an ATP-independent manner, was added to the motility assay solution instead of G680V HMM, the movements of the filaments were not accelerated, and were halted when the concentration of NEM-S1 exceeded 0.1 μM (Fig 3A and 3C). This result suggests that a rigor-like, strong interaction between actin filaments and the myosin motor domain is unable to accelerate the sliding movement. One may wonder if NEM-S1 in solution also has accelerating effects, but at the same time, exert slowing effects by connecting moving actin filaments with the nitrocellulose surface. This is unlikely because, under our blocking conditions with 30 mg/mL BSA, skeletal muscle HMM was unable to bind to the surface. Native skeletal muscle S1 molecules in the motility assay solution were also unable to accelerate these movements (Fig 3B and 3C). G680V mutant myosin was originally isolated on the basis of cold-sensitive *in vivo* myosin functions in a genetic screening using *Dictyostelium* cells [19], so it should have some residual motor activities at warmer temperatures, including at 26°C, which was used in this study. Biochemically, G680V mutant myosin has actin-activated ATPase activity that is roughly half of that of wild type myosin, with very low *K*_*app*_ for actin and very slow phosphate and ADP release rates [19, 20, 26]. Therefore, unlike wild type myosin II motors, which are mostly detached from actin filaments under standard ATPase conditions, the majority of G680V myosin motors are bound to actin filaments with an intermediate affinity in either the state of actin-myosin-ADP-Pi or actin-myosin-ADP [20]. We thus speculate that the abundance of either the actin-myosin-ADP-Pi or actin-myosin-ADP state, and not the rigor-like complex involving NEM-S1 or transient interaction with native S1, is essential for the acceleration of these movements. Further biochemical and structural studies are clearly needed to elucidate how the actin structure is affected by G680V heads.

### Incorporation of acto-G680V S1dC chimera molecules into actin filament

In the third experiment, we used a chimeric protein that is based on acto-S1dC, which consists of *Dictyostelium* S1 lacking the light chain binding sites (S1dC; [27]) carrying an entire *Dictyostelium* actin sequence inserted in loop2, one of the actin binding sites [23]. Acto-S1dC is a mimic of actin and S1dC crosslinked at the native interaction site, so that it is able to copolymerize with skeletal muscle G-actin. When copolymerized, the S1dC portion of the chimeric protein hydrolyzes ATP at about one third of the rate of Vmax of native S1dC. In this study, we introduced the G680V mutation into the S1dC moiety of the acto-S1dC gene, and prepared the recombinant protein as described previously [23].

When skeletal muscle G-actin was copolymerized with 1/30 (mol/mol) acto-G680V S1dC, the sliding velocity of the copolymers on skeletal muscle HMM surfaces again increased about two fold (Fig 4), supporting the above conclusion that anchoring of the G680V myosin motor to the substrate is unnecessary for faster movement. If the acto-G680V S1dC molecules homogeneously copolymerized with skeletal muscle G-actin, then a naïve prediction based on the fact that acceleration was maximum at a molar ratio of 1:30 is that the conformational changes in actin filaments to accelerate the sliding movements are propagated, on average, 30 actin protomers or one helical pitch, originating from one G680V acto-S1dC molecule incorporated in the copolymer. However, electron microscopic observation of the copolymer (Fig 5) demonstrated that the number of S1dC moieties was much less than expected from homogenous copolymerization of acto-G680V S1dC and skeletal muscle G-actin, i.e., one per helical turn. This may indicate that acto-G680V S1dC and skeletal muscle G-actin do not copolymerize efficiently. If this is the case, a molar ratio of acto-G680V S1dC to skeletal actin in copolymers lower than 1:30 is sufficient for maximum acceleration, implying that a single acto-G680V S1dC molecule affects much more than 30 neighboring actin protomers.

Copolymers containing higher concentrations of acto-G680V S1dC either moved slowly, stopped movement or diffused away from the surface. One simple explanation for this is that the lower fraction of skeletal muscle actin protomers in the copolymers would decrease the density of available binding sites for skeletal muscle HMM on the surface, leading to slower movement or detachment of the filaments from the surface. The same explanation may be applicable to the deceleration of movements in the presence of a high concentration of G680V HMM in the motility assay solution.

In this report, we have provided further evidence that the conformational state of single actin filaments plays important roles in the determination of sliding motility on skeletal muscle HMM motors, and that interactions of actin filaments with myosin motor carrying ADP or ADP and Pi accelerate sliding movements.
